# E.Co.Tech-electrochemical handheld breathalyzer COVID sensing technology

**DOI:** 10.1038/s41598-022-08321-x

**Published:** 2022-03-14

**Authors:** Ivneet Banga, Anirban Paul, Kordel France, Ben Micklich, Bret Cardwell, Craig Micklich, Shalini Prasad

**Affiliations:** 1 Department of Biomedical Engineering, University of Texas at Dallas, 800 W Campbell Rd., Richardson, TX 75080 USA; 2 Sotech Health, 17217 Waterview Pkwy, Dallas, TX 75252 USA; 3 Cleveland Clinic Abu Dhabi, Al Maryah Island, Abu Dhabi, United Arab Emirates

**Keywords:** Biomarkers, Diseases, Medical research, Chemistry, Engineering, Nanoscience and technology, Biotechnology, Nanobiotechnology

## Abstract

Breathomics is widely emerging as a strategy for non-invasive diagnosis of respiratory inflammation. In this study, we have evaluated the metabolic signals associated with Coronavirus (SARS COV-2), mainly the release of nitric oxide in breath. We have demonstrated the utility of a breath analyzer-based sensor platform for the detection of trace amounts of this target species. The sensor surface is modified with Room Temperature Ionic Liquid (RTIL) that allows faster diffusion of the target gas and can be used for gas sensing application. A low limit of detection (LOD) of 50 parts per billion has been achieved with a 95% confidence interval for detection of nitric oxide.. This inhouse designed sensor is incorporated into a breath analyzer system that displays enhanced sensitivity, specificity, linearity, and reproducibility for NO gas monitoring. The developed sensor platform can detect target concentrations of NO ranging from 50 to 250 ppb, using 1-Ethyl-3-methylimidazolium Tetrafluoroborate ([EMIM]BF_4_) as RTIL and displays fast response time of 5 s, thereby allowing easy detection of the target gas species. The sensor successfully quantifies the diffusion current and charge modulations arising within the electrical double layer from the RTIL–NO interactions through DC-based chronoamperometry (CA). The subjects tested negative and positive are significantly different (p < 0.01). The prototype can potentially be used for human health monitoring and screening, especially during the pandemic due to its portability, small size, an embedded RTIL sensing element, integrability with a low-power microelectronic device, and an IoT interface.

## Introduction

Coronavirus, a family member of single stranded RNA viruses consists with potent viral genome, covered by a bilayer lipidic envelope and a large number of peplomers or spikes on the surface, is currently considered the scariest health hazard worldwide with its potent ability to spread and infect human mankind never than before. There are many different variants of it, and most of their variants spread rapidly and cause infection at human respiratory tract, which ultimately leads to serious respiratory illness, resulting in extremely high morbidity and even death. The global COVID-19 pandemic is caused by the spread of severe acute respiratory syndrome coronavirus 2 (SARS CoV-2)^1–4^. Nearly 372 million people have tested positive worldwide and nearly 5.6 million people have lost their lives due to this deadly outbreak till February 2022. The virus spreads via airborne transmission, that is droplets, blood borne transmission and fecal–oral transmission^5^. One of the important factors regarding rapid spread of this disease includes its general symptoms being confused with common flu. Scientists at the Centers for Disease Control (CDC) and World Health Organizations have identified common symptoms of this disease including dry cough, headache, difficulty breathing, weakness, and lack of smell and taste. Moreover, it has been found in some extreme cases, dyspnea and/or hypoxemia can occur a week after the appearance of the disease, accompanied by septic shock, acute respiratory distress syndrome (ARDS), and dysfunction of coagulation^6^. These different modes exacerbated the rapid spread of the virus and made it highly difficult to control the spread. A person infected with the COVID-19 virus can display symptoms such as fever, cough, or shortness of breath that can be completely non-specific to one disease or can remain asymptomatic. Even during the incubation period of the virus, the person infected can still transmit the virus particles to others^7^. This pandemic shows no sign of ending due to the recent mutations of the novel coronavirus^8^. The only way to control the spread of this pandemic is via rapid diagnostic testing and tracing. The gold standard test for the diagnosis of COVID-19 is reverse transcription–polymerase chain reaction (RT-PCR) and quantitative RT-PCR (qRT-PCR). These tests, which involve viral detection, are highly sensitive as well as specific^9,10^. There are multiple occasions when RT-PCR also fails to quantify this disease due to several technical reasons, although patients show strong symptoms. Along with RT-PCR, chest X-ray, radiology and computed tomography (CT) is also currently considered one of the alternate gold standards for the detection of this novel coronavirus and in most cases such procedures have been opted if there is a confusion from RT-PCR^11^. However, these tests are time consuming, relatively expensive, require sophisticated instrumentation, and trained personnel^9,10^.

Current research shows that COVID-19 has been strongly associated with pulmonary deficits^12^. Key metabolic signals associated with tracking the pulmonary deficit are a combination of volatile organic compounds (VOCs) and inorganic gases. These VOCs and gases are found in parts per billion to parts per million levels in human exhaled breath. Exhaled breath can be used for non-invasive disease diagnosis, the term being used widely for such an approach is depicted as “breathomics”. VOCs from exhaled breath can arise from cellular in vivo metabolic activity. Respiratory diseases often alter metabolic pathways such as lipid peroxidation and upregulate the release of cytochrome P450 enzyme^13^. This altered metabolic pathway releases VOCs in the breath, which can be linked to specific respiratory pathways. Target analytes such as nitric oxide (NO) and methanol have also been found in human breath using resistance-based sensors. In a study, it was found that COVID-19 patients with thrombotic complications had higher NOX activation compared to event-free ones, independently upon confounders such as coexistent atherosclerotic burden including coronary artery disease and peripheral artery disease and age^14,15^. In a study performed by Violi et al.^16^, their hypothesis is supported by NOX2 overactivation in COVID-19 patients with > 40% increase compared to controls. The study provides evidence that compared to controls, COVID-19 patients display overactivation of NOX2, which is more marked in patients admitted to ICU. In another study, it was validated that activation of NOX genes increases by the release of NO by the human endothelial cells^17^. These results support our hypothesis, and we believe that detection of these biomarkers in an array-based manner will help improve the sensitivity and specificity of disease diagnosis. Current technique being used for the detection of VOCs is based on gas chromatography mass spectroscopy (GC–MS), which requires sophisticated instrumentation and prohibits point of care application. Furthermore, it is especially important that the time course of appearance of these biomarkers of inflammation roughly coincides with the time course of disease symptoms, especially onset. Regardless of the instrumentation used to detect these biomarkers, measurements must be made repeatedly on definitively or potentially infected individuals to map the rise and fall of the biomarkers over time.

Electrochemical point of care devices are popular for their effectiveness, ability to interface with Internet of Things (IoT) devices, and ease of access toward various applications^18–22^. These devices fundamentally operate on electroanalytical principle, where electrode electrolyte interface is explored to obtain signal response as a function of potential/current. These devices are also known as electrochemical sensors mainly classified in two types: electrochemical chemo sensors and electrochemical biosensors. Electrochemical biosensors are mainly electrochemical devices which are able to capture biological/biochemical recognition and are able to transduce the signal using suitable electroanalytical tools. There are numerous electrochemical biosensors reported in literature using various materials, used for signal transduction^23–25^. Room Temperature Ionic Liquids (RTILs) are widely employed as a sensing material for surface modification of electrodes to allow sensing of various gases and VOC^26–28^. We have demonstrated the use of RTIL as a signal transducer for the detection of trace VOCs and gases^21,29,30^. Moreover, the current breath analyzer-based technology developed for COVID-19 detection include magnetic sensors^31^, optical sensors^32^ and electrochemical sensors^33^. They suffer from drawbacks such as lack of portability into a handheld device, long detection time and interference to signal^34^.

**Scheme 1:**
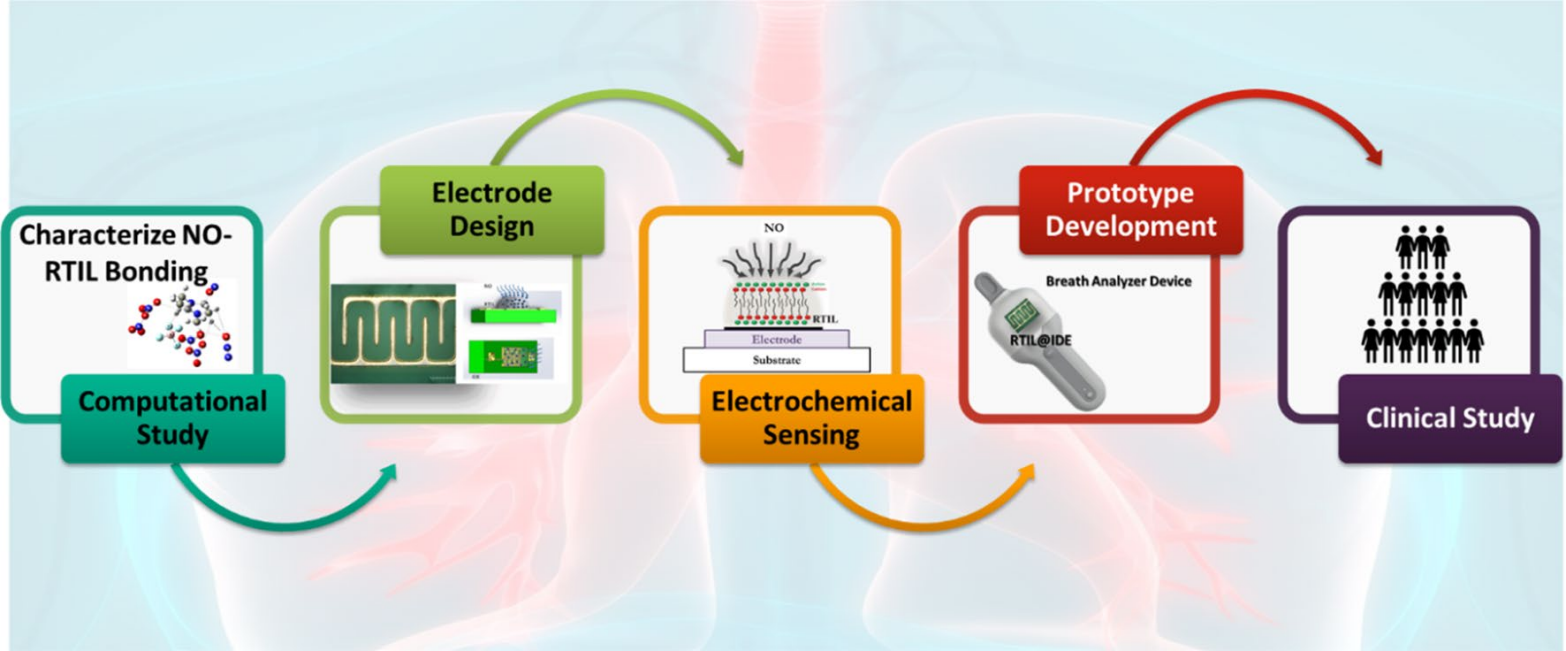
Schematic representation of the experimental sensing steps used for prototype development and characterization of NO–RTIL interaction.

In this paper, we have demonstrated the first of its kind breathomics device for the detection of NO as a potential biomarker for COVID-19 (Scheme 1). We have demonstrated the utility of a breath analyzer-based sensor platform for the detection of trace amounts of this target species. The sensor surface is modified with RTIL that allows faster diffusion of the target gas and can be used for gas sensing application. This inhouse-designed sensor platform can be used for the measurement of NO with concentrations as low as 50 ppb. We used a potential of 1 V to perform chronoamperometry (CA). This specific potential allows maximum diffusion of the target analyte, in this case NO. The change in current for the target analyte from baseline is three times greater than the baseline and statistically different from cross-reactive species (p ≤ 0.01). We performed cross-reactive studies for other target analytes such as carbon dioxide, acetone, and methanol that are often found in the breath because of respiratory diseases. We have also evaluated the metabolic signals associated with coronavirus (SARS COV-2) using the in house-designed breath analyzer platform. A total of 81 human subjects have been tested, both in different use settings and within the infection cycle. The results so obtained have been statistically analyzed and reported in the paper.

## Results and discussion

The organization of this section is as follows: (1) COMSOL™ simulation explaining the rationale behind the use of interdigitated electrode design, (2) Computational study to select EMIM[BF_4_] as a suitable candidate for NO sensing, (3) Characterizing the NO binding interaction with EMIM[BF_4_] for electrochemical gas sensing, (4) Translatability of the RTIL–NO interaction toward low power portable microelectronic prototype development, (5) Validation of the low power portable prototype for real-time NO detection in a clinical setting.

### COMSOL™ simulation explaining the rationale behind using the interdigitated electrode design

**Figure 1:**
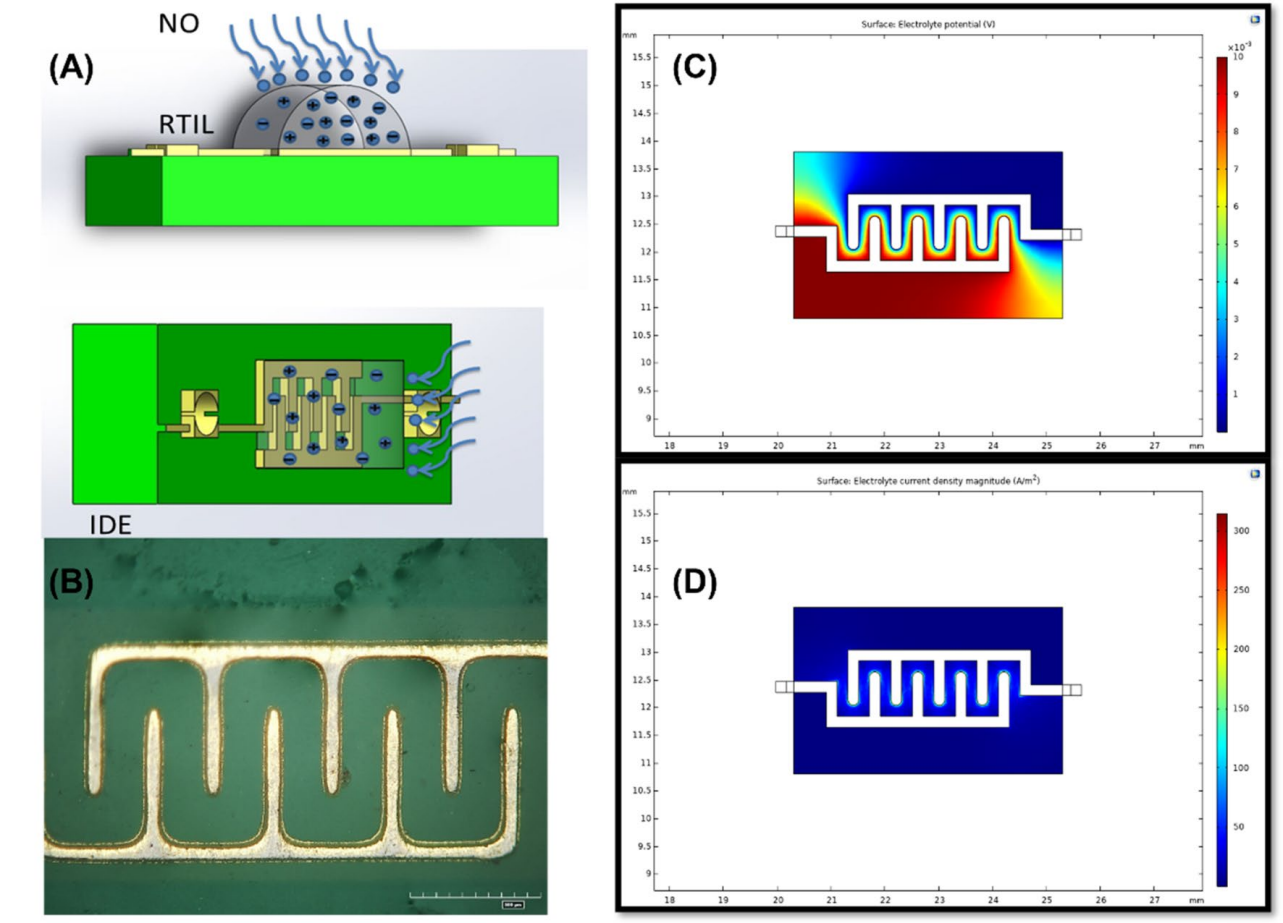
(**A**) Schematic of the IDE modified with RTIL and NO sensing (using Microsoft PowerPoint). (**B**) Microscopy image of the modified electrode (HIROX microscope). (**C**) COMSOL simulation (https://www.comsol.com/) representing electrolyte potential for working and reference electrodes. (**D**) COMSOL simulation (https://www.comsol.com/) representing electrolyte current density for working and reference electrodes.

For electrochemical sensing application, we use an interdigitated electrode design (Fig. 1A,B) to develop a planar capacitive sensor using RTIL as the transducer. An interdigitated electrode (IDE) offers advantage for gas sensing as it allows increased signal response due to electric field confinement. It helps in capturing the change in dielectric permittivity upon diffusion of the target gas. Moreover, gold is appropriate as the electrode material since it is electrochemically stable and possesses chemical inertness. We performed COMSOL (https://www.comsol.com/) simulation to replicate the electrode-electrolyte interface. Electrical parameters are applied to both the working electrode and the reference electrode (Fig. 1C,D). A constant AC potential of 10 mV with a DC bias is applied to the WE with respect to the RE to obtain enhanced sensing performance; the RE is grounded/insulated. The electric fields are confined within the RTIL–electrode interface boundaries. The electrolyte potential is maximum at the digits of the WE and varies from 0.01 to 0.001 V moving from WE to the RE in a smooth gradient manner for an input bias of 10 mV. Maximum current density is observed at the WE which influences the output response of the system. The surface current densities (Fig. S1) contributed by the electrode-electrolyte interface is dominant around the WE and equated to 289.83 A/m^2^. This surface decays with a transient angle from WE to RE (as expected for a two-electrode system with no additional counter electrode) with no parasitic currents contributed by the electrodes going from WE to RE.

### Computational study to select EMIM[BF_4_] as a suitable candidate for NO sensing

**Figure 2:**
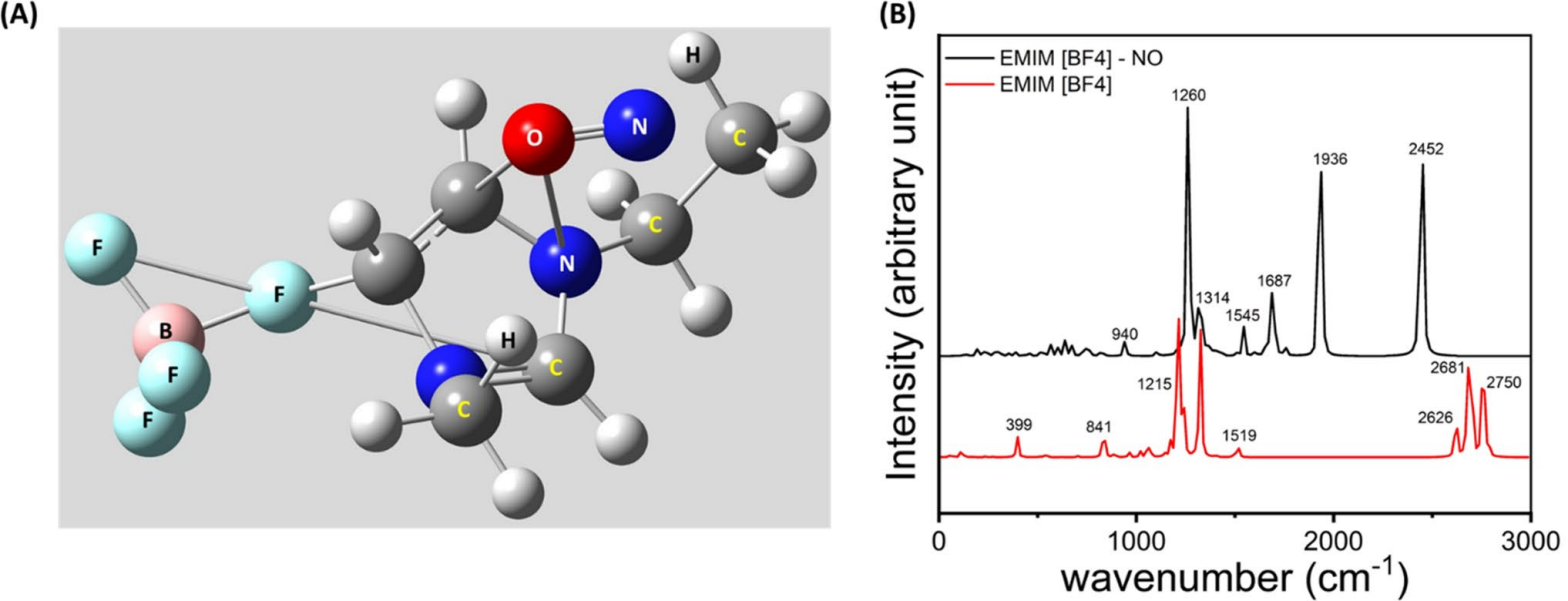
(**A**) Optimized structure of EMIM[BF_4_]-NO depicts strong interaction of NO with EMIM[BF_4_] moiety. (**B**) FTIR comparison of EMIM[BF_4_] and EMIM[BF_4_]-NO showing substantial peak shift.

A computational study has been done to visualize the interaction of the RTIL and NO. For this purpose, the structure of the RTIL is optimized using Hartree Fock, having a basis set of 6–31 g (d). The optimized structure of EMIM[BF_4_] is depicted in Fig. S2. The optimized structure of EMIM[BF_4_] suggests that there is a strong ionic interaction between EMIM^+^ and BF_4_^−^ which makes this species stable. Then the RTIL NO interaction has been visualized by optimizing EMIM[BF_4_] and NO. The optimized structure of EMIM[BF_4_]-NO has been depicted in Fig. 2A. The result suggests the presence of strong non-covalent interaction between the O atom of NO and C3 and N1 of the imidazolium ring, which implies that the RTIL has strong affinity towards NO. As EMIM[BF_4_] is an ionic species present in zwitter ionic form, it has the ability to interact with a variety of species having positive or negative polarity. We have also calculated the HOMO–LUMO energy of EMIM[BF_4_], NO and EMIM[BF_4_]-NO, depicted in Table 1.

**Table 1:** Calculated HOMO–LUMO energy of the RTIL, NO and RTIL–NO.

Compound	E_HOMO_ (Hartree)	E_LUMO_ (Hartree)
EMIM[BF_4_]	− 0.40587	− 0.03340
NO	+ 0.02623	+ 0.36548
EMIM[BF_4_]-NO	− 0.03848	− 0.13730

We have calculated the HOMO–LUMO energy gap of RTIL and NO.

$$ \left({\text{E}}_{{{\text{HOMO}}}}^{{{\text{EMIM}}\left[ {{\text{BF}}_{4} } \right]}} - {\text{E}}_{{{\text{LUMO}}}}^{{{\text{NO}}}} \right) - \left({\text{E}}_{{{\text{LUMO}}}}^{{{\text{EMIM}}\left[ {{\text{BF}}_{4} } \right]}} - {\text{E}}_{{{\text{HOMO}}}}^{{{\text{NO}}}} \right) = ( - 0.{4}0{587} - 0.{36548}) - \, ( { - 0.0{334}0 - 0.0{2623}} ) = \, - 0.{77135} + 0.{36}0{23} = \, - 0.{\text{41112 Hartree}} $$

$$ \left (E_{HOMO}^{{EMIM\left[ {BF_{4} } \right] - NO}} - E_{LUMO}^{{EMIM\left[ {BF_{4} } \right] - NO}} \right) = - 0.03848 - ( - )0.13730 = - 0.0{9882 }( {{\text{Hartree}}} ) $$

From the calculation, it has been found that the HOMO–LUMO energy gap has been substantially reduced (~ 30%) after the formation of EMIM[BF_4_]-NO. This suggests that the interaction is feasible. We have also pulled out the thermodynamics data which shows that the electronic + thermal free energy of EMIM[BF_4_]-NO is − 0.440047 Hartree whereas the electronic + thermal free energy of EMIM[BF_4_] is − 0.421756 Hartree, which suggests the formation of EMIM[BF4]-NO is indeed thermodynamically feasible too.

We have also obtained the theoretical FTIR data of EMIM[BF_4_]-NO and EMIM[BF_4_] and the result is presented in Fig. 2B. The result shows standard FTIR peaks of EMIM[BF_4_] at 841 cm^−1^ depicting C–H bending, 1215 cm^−1^ C–H stretching. Most other peaks are related to standard C–H stretching and bending, EMIM ring stretching etc. A substantial peak shift has been observed for EMIM[BF_4_]-NO which suggests substantial interaction is taking place. This result leads us to carry forward our sensing mechanism to the next level.

### Characterizing the NO binding interaction with EMIM[BF_4_] for electrochemical gas sensing

RTILs are a new class of materials that can be utilized as a low-power, easy maintenance solution to develop a portable gas sensing system. RTIL’s are solvent free electrolytes consisting of cation/anion pairs. RTILs possess unique physicochemical properties such as high ionic conductivity, low volatility, high thermal stability, and wide electrochemical window which are advantageous from the perspective of gas sensing. All the electrochemical experiments have been carried out using an optimized DC potential of + 1.0 V. Experiments were performed under controlled N_2_ flow. CA has been performed at three different potentials and chronoamperogram were plotted at 5 s for three replicates. We have optimized the operating potential to be + 1.0 V and time window to be 5 s as the interquartile range of variation is small and data is consistent for all replicates (Fig. S3).

**Figure 3:**
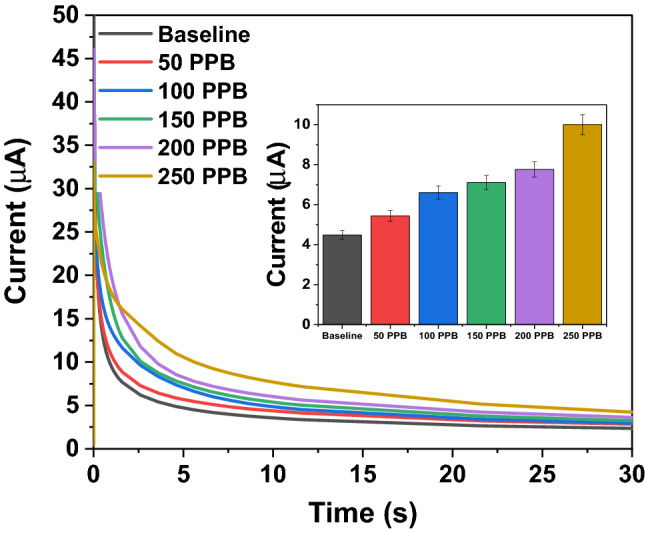
Chronoamperometry scan was performed at + 1 V for 30 s on the EMIM[BF_4_] modified interdigitated electrode (IDE) for the target analyte. Calibrated dose response chronoamperogram for NO concentration ranging from 50 to 250 ppb.

Calibrated dose response is the most important characteristic which depicts the performance of the sensor. Dose-dependent response was studied using five different variations of NO concentration for the developed device, having the sensor attached. NO has been mixed with N_2_ volumetrically to obtain four NO dilutions—50 ppb, 100 ppb, 150 ppb and 250 ppb. We have selected this sensing range according to the exhaled nitric oxide levels and their correlation to the underlying respiratory inflammation^35^. Chronoamperometry is used to characterize the diffusion limited behavior of the RTIL modified electrode system. The potential was applied at 1 V for 30 s and the current at 5 s was extracted and plotted in the inset of Fig. 3.

The modified electrode–electrolyte interface acts as a semi-permeable layer, which creates a concentration gradient at the interface to allow easy diffusion of the target gas. The equation for Fick’s law of diffusion is as follows:

1 $$ {{J}} =  - {{D}}\frac{{\partial {{C}}}}{{\partial {{X}}}} $$

where J is the diffusion flux, D is the diffusion coefficient, x is the position, and C is the concentration.

For studying the diffusion characteristics using chronoamperometry as the transduction principle, we use the Cottrell equation as follows:

2 $$ {{i}} = \frac{{{{nFAc}}_{{{j}}}^{0} \sqrt {{{D}}_{{{j}}} } }}{{\sqrt {{\pi t}} }} $$

where, *i* is the current due to diffusion, $c_{j}^{0}$ is the concentration of the diffused species, and *t* is time.

Chronoamperometric measurement records the cathodic current generated due to the diffusion of the NO molecules on the electrode–electrolyte interface. The transient cathodic diffusion current at 5 s with increasing concentration has been plotted in the inset. This transient current depicts the dynamic gas diffusion phenomenon across the electrode–electrolyte interface before the system reaches a steady state. Logarithmic scale fitting (log 3P1) was performed for the calibrated dose response and an R^2^ value of 0.96 was obtained for the entire concentration range.

### Evaluation of EMIM[BF_4_] @IDE modified sensor specificity and selectivity for NO sensing in the presence of cross-reactive gases

**Figure 4:**
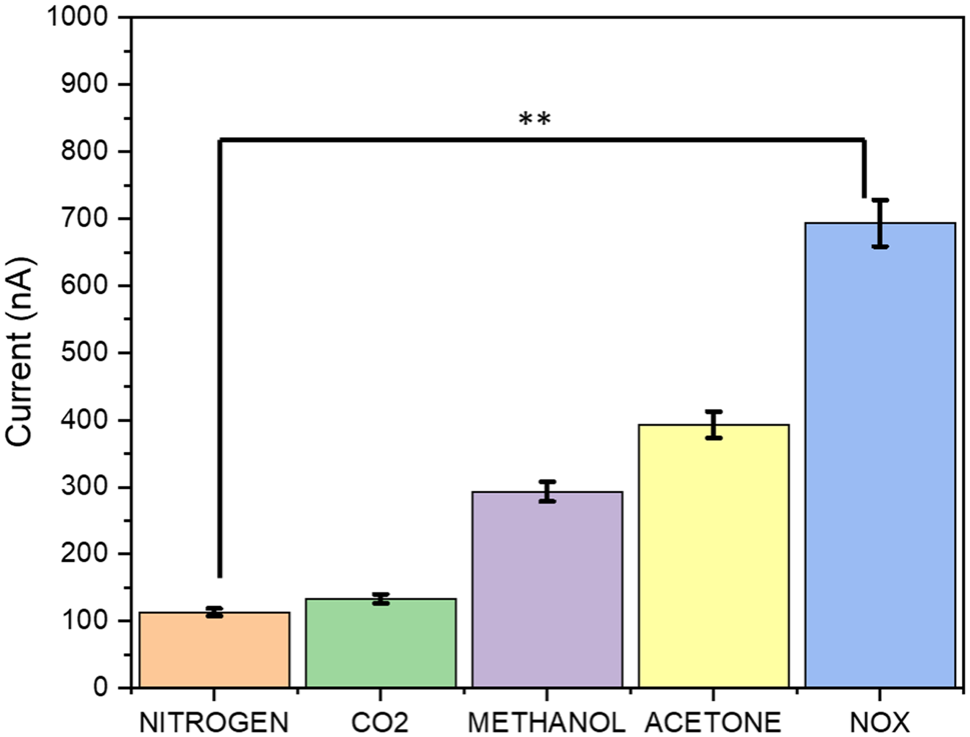
Selective sensing response of the modified electrode–electrolyte interface toward the detection of other gases and volatile chemical compounds, including nitrogen, carbon dioxide, methanol and acetone as compared to selective sensing for NO target gas.

We evaluated the selectivity and specificity of the modified electrode–electrolyte interface toward electrochemical sensing of NO over other environmental gases and VOCs that are present as cross-reactive molecules. The selectivity performance of NO at 100 ppb over nitrogen, carbon dioxide, methanol, and acetone are shown in Fig. 4. The fabricated sensor platform displays a distinguishable and specific CA response for the detection of NO. The average transient diffusion current at 5 s and 1 V was plotted, and it was observed that the average nonspecific signal for cross-reactive gases and vapors was 100 nA, whereas the specific signal for the detection of NO was 700 nA, which was more than ~ 3 times larger than the nonspecific signal.

### Translatability of the RTIL–NO interaction toward low power portable microelectronic prototype development

**Scheme 2:**
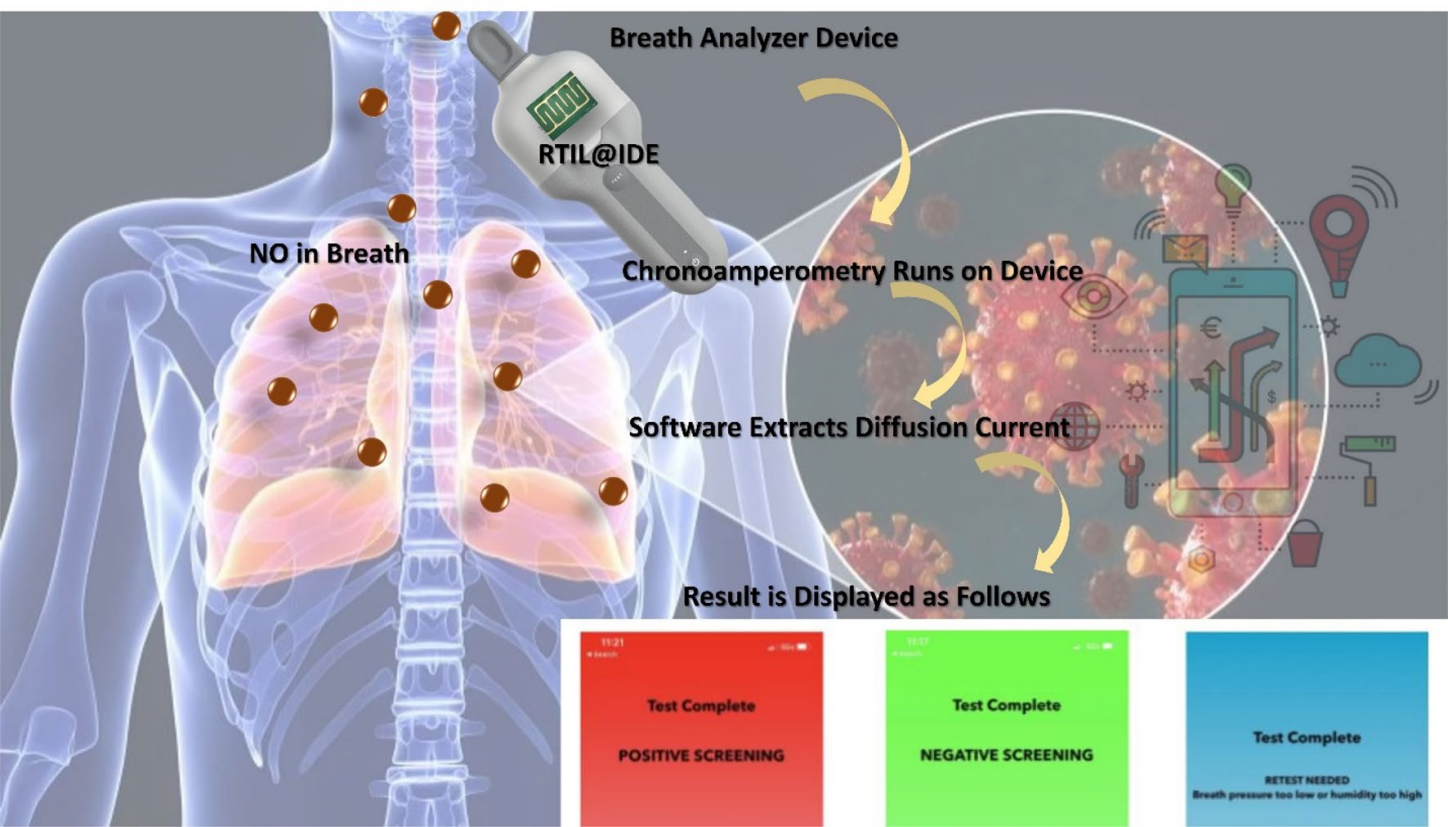
Working scheme of the prototype development consisting of RTIL@IDE connected to low power controller and the steps followed post breath collection.

COVID-19 is a humanitarian crisis. Governments, healthcare systems, regulatory agencies, research institutes, and industry players are working together to understand and address the challenge, support victims and their families and communities, and search for treatments and a vaccine. We believe that having an effective and rapid “decision support” device to complement existing diagnostic tests would enable much better containment of the virus. In consideration of these points, we developed a portable NO sensing prototype device in the form of a breath analyzer (see Supplementary Video SV1). The device leverages two microcontrollers that work collaboratively—one acting as a master controller to coordinate driver functions of the device and the other as a slave controller performing the aforementioned electrochemical analysis. The slave microcontroller performs computation on data received from the end-effector, which in this case is a sensor with an IDE coated with RTIL measuring EMIM[BF_4_] as the target analyte (Scheme 2). The top end of the device has a single-use mouthpiece that is discarded after every subject breathes into the device. The device contains a slot near the top designed for a single-use mouthpiece that is discarded after every subject breathes into the device. The user initiates the electrochemical analysis sequence by pressing a “Test” button on the device itself. Upon initiating this sequence, the user is instructed to wait while the device begins a Chronoamperometry sequence in the manner described above and assesses baseline values of the target analyte as it exists in the current ambient environment. This sequence can be denoted as the “baseline sequence” and its results are stored locally for reference. When the baseline sequence terminates, the user is instructed to exhale into the device via the mouthpiece. Simultaneously, the device begins another identical Chronoamperometry sequence that assesses values of the target analyte over the interpreted breath as it passes over the sensor. We denote this sequence as the “stimulus sequence”. Six seconds are allotted for the breathing window over this stimulus sequence, but only three seconds are necessary in order to obtain an adequate result. Upon completion of the stimulus sequence, several algorithms present within the device firmware extract the 6-s diffusion current value from the stimulus sequence and compare it to the results similarly obtained by the baseline sequence. Note that, during both the baseline and stimulus sequences, several other sensors perform local measurements of the environment around the sensor IDE. Their data are also contributors to the final test readings, and we discuss these other sensors in the following section. At this point, the assessment is considered complete, but an optional step may be employed to wirelessly transmit assessment results to a database for storage or to a mobile application for easier visualization.

**Figure 5:**
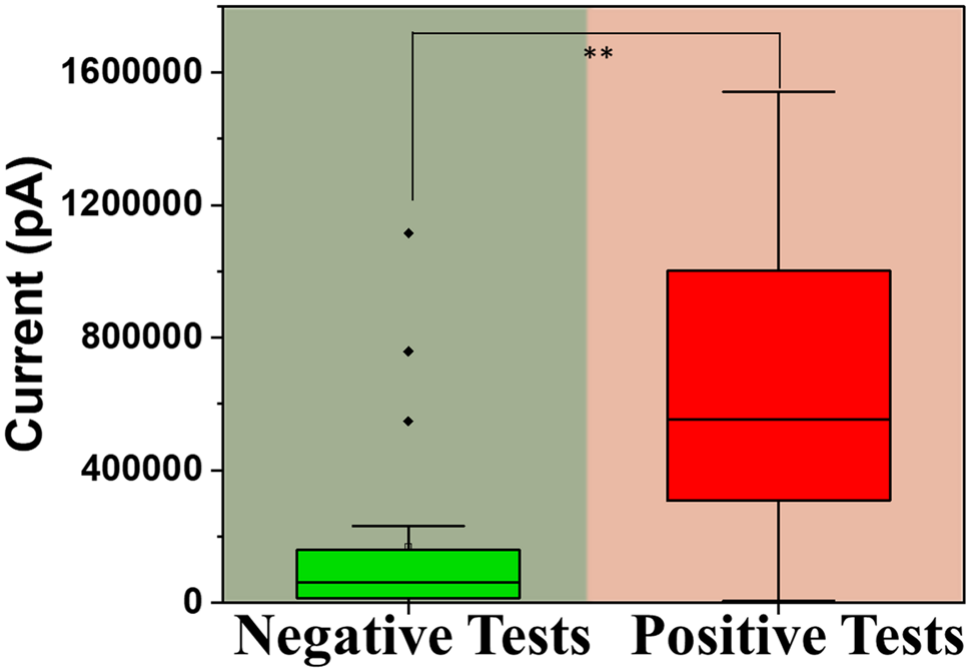
Box plot representing maximum measured current (nA) obtained during the breathing cycle as obtained using the device for both the positive and negative tests.

In this study, we evaluated the metabolic signals associated with coronavirus (SARS COV-2) such as up regulation of NO levels in breath. 84 human subjects have been tested, both in different use settings and within the infection cycle. It was found that the breath analyzer is superior to other techniques used for screening purposes. We believe that diagnostics tests do have an important role as “positive confirmatory test” and/or “negative confirmatory test when the clinical symptoms are not aligned with screening test outcome”. The signal obtained is first depicted as raw current value. Figure 5 represents the signal recorded by the breath analyzer device for all the patients. The graph depicts maximum measured current (pA) obtained during the breathing cycle. The subjects tested negative and positive are significantly different (p < 0.01).

**Figure 6:**
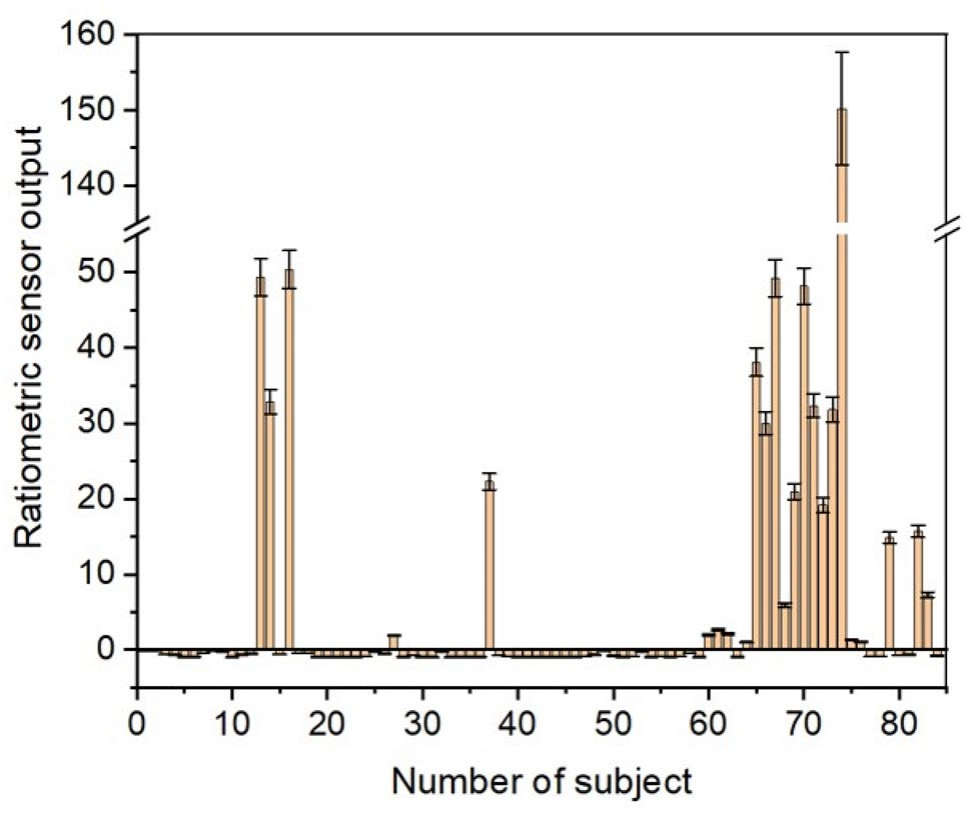
Ratiometric sensor output as recorded by the breath analyzer device for 84 subjects. The response is plotted as a ratio metric signal wherein in the signal is compared to the baseline.

Moreover, the data obtained for the breath sample collected from 84 human subjects was plotted as ratiometric sensor output where the ratio is calculated for the patient signal response with respect to the baseline (Fig. 6). A positive ratio provides strong evidence to support a COVID-positive subject while a negative ratio provides strong evidence to support the absence of the disease. A positive signal is the current value is greater than the threshold value set, therefore has a positive change in current. On the other hand, negative signal is the current value that is below the threshold set for the sensing NO, therefore has a negative change in current.

The breath analyzer determines a positive or negative result through a combination of weighted parameters, including both measured electrical responses, sample pressure and humidity. On the surface, with all other parameters held constant, an electrical charge of specific magnitude that occurs across the RTIL above a certain threshold is indicative of the presence of the virus in a patient’s body when a breath sample of the patient is analyzed by the device. However, there are several environmental variables that influence this response in a real-world application such as pressure, relative humidity, and temperature of both the patient’s breath sample and the surrounding ambient air that make this determination of the inorganic gas and metabolite concentration not so simple. The electrical current threshold mentioned above is subtle and easy to mistake if these parameters are not accounted for. Due to this, these devices contain several sensors that monitor the electrical charge across the RTIL along with the air pressure, relative humidity, and temperature of both the ambient air and the breath sample received from the patient. An algorithm present on the device takes these variables as inputs and passes them through several de-noising filters to maximize the signal-to-noise ratio. Due to the parameters that need to be accounted for, the data passes through these filters to extract the true normalized electrical signal. The slope, convexity, and final level of this signal are then analyzed to determine whether the breath sample contains a concentration of the specific metabolite and inorganic gas above the threshold. This algorithm was initially trained empirically and fine-tuned through accumulated experimental and clinical data. The algorithm uses laws of gas dynamics as well as other principles observed through internal experimentation on accumulated data. The statistical parameters as calculated by the data generated using the human subject-based testing have been summarized in Table 2.

**Table 2:** Statistical parameters describing the efficiency of the breath analyser.

Statistical performance parameter	Value (%)
Sensitivity	100
Specificity	87.04
Accuracy	91.36
True positive	100
True negative	87.04
False positive	12.96
False negative	0.00

Hence, this work is a first-time demonstration of a portable, low-power microelectronic platform for rapid and dynamic detection of NO levels in exhaled breath making it a suitable device for use in screening for the presence and absence of COVID-19. We have summarized the materials/methods/techniques that have been used for COVID-19 sensing along with our work (Table S1).

## Conclusion

Breath based analysis is being widely employed for the development of point-of-care devices for non-invasive disease detection. Breath analytics is based on the detection of volatile organic compounds and inorganic gases that are released endogenously because of the metabolic pathways. The concentration of these metabolites varies due to alteration in the endogenous metabolic pathways and can be correlated to understand the underlying disease condition. This work is a first-time demonstration of a portable, low-power microelectronic breath analyzer for rapid and dynamic detection of nitric oxide in exhaled breath for coronavirus screening. A computational study is adapted towards development of an electrochemical sensor platform. The sensor surface is modified with RTIL that allows faster diffusion of the target gas and can be used for gas sensing application. This inhouse-designed sensor platform can be used for the measurement of NO with concentrations as low as 50 ppb. The developed sensor platform can detect target concentrations of NO ranging from 50 to 250 ppb, using 1-Ethyl-3-methylimidazolium Tetrafluoroborate ([EMIM]BF_4_) as RTIL and displays fast response time of 5 s, thereby allowing easy detection of the target gas species. The sensor successfully quantifies the diffusion current and charge modulations arising within the electrical double layer from the RTIL–NO interactions through DC-based chronoamperometry. The subjects tested negative and positive are significantly different (p < 0.01). The prototype can potentially be used for human health monitoring and screening, especially during the pandemic due to its portability, small size, an embedded RTIL sensing element, integrability with low-power microelectronic device, and an IoT interface.

## Materials and methods

### Reagents and materials

Ionic liquid such as 1-Ethyl-3-methylimidazolium Tetrafluoroborate ([EMIM]BF_4_) ≥ 99.0% (HPLC) was procured from Millipore Sigma. A Trace Source disposable permeation tube for nitric oxide was procured from KINTEK Analytical. All safety precautions were followed while working with nitric oxide gas. The gas mixing system was set up inside the fume hood, and the residual gas after electrochemical sensing was discarded using a bubbler system. Proper protective equipment and all safety precautions were always followed while running experiments with the toxic gas to avoid skin and eye contact or accidental inhalation and digestion of the gas.

### Electrode design and material

The electrode sensor design employed in this study has previously been used by our group for studying amperometric gas sensing phenomenon^29,30,36–38^. A gold interdigitated electrode (IDE) design was chosen for sensing of target gas analyte as gold offers increased electric field confinement owing to the reduced microelectrode geometry. The design parameters such as the spacing between the digits and the digit size was determined by COMSOL simulation. The spacing within the digits was optimized at 0.2 mm, width and length of the digits was fixed at 0.2 mm and 0.8 mm respectively. Custom designed IDEs were procured from PCB Way. Prior to each experiment, the sensor was first soldered for ease of connection to Gamry potentiostat. The surface of the sensor was cleaned with acetone, isopropyl alcohol and then finally dried with N_2_ gas. 3 µL of the RTIL was drop casted onto the sensor surface and was used for gas sensing experiments.

### Electrochemical characterization of surface modified sensor

The electrochemical window optimization for the surface modified IDE was done at 0.8 V, 1 V and 1.2 V. Experiments were performed under controlled N_2_ flow. Chronoamperometry was performed at three different potentials and chronoamperograms were plotted at 5 s for three replicates. Once the electrochemical potential was optimized, the next step involved determination of the signal to noise ratio for the baseline and breath sensing current. Double potential chronoamperometry was performed at 1 V for 30 s and − 1 V for 30 s. Transient diffusion current at 5 s was extracted and plotted to understand the difference between the baseline measurement and signal for the target gas. Furthermore, cross reactivity study was performed to determine the sensor specificity towards target gas sensing by performing chronoamperometry at 1 V for 30 s. The study was performed to analyze sensor response towards nitrogen, carbon dioxide, methanol, acetone, and NOx. The current at 5 s was plotted and compared. The Gamry Series 600 potentiostat/galvanostat was used to perform electrochemical characterization and gas sensing. All safety considerations were followed while handling target gas analyte.

### Translatability of the RTIL–NO interaction toward low power portable microelectronic breath analyzer development

The initial study provided many opportunities for optimization of the device, namely for usability, accuracy, and technical advantage. While the prototype Breath Analyzer performed screening of the target analyte successfully, several improvements helped evolve the prototype into a more robust device that was more versatile in use. The sensor, as with all microelectronic sensors, is subject to drift effects over prolonged periods of activity. Since the resolution of sensor measurements are in pA (one trillionth of an ampere), this issue is further exacerbated due to inherent noise present in other sensors within electronics assembly. Software algorithms were employed to help compensate for these drift effects and maximize the signal-to-noise ratio (SNR). Additionally, the ionic liquid-electrode interface relaxes over subsequent tests, reducing the capacitance of the sensor if proper electrochemical sequencing is not employed to preserve it, so extensive laboratory and field testing was performed to determine the optimal sequence parameters. For example, open circuit potentiometry (OCP) analysis was proven to provide a great indication of the capacity of the RTIL before a test, so a short OCP sequence was employed to occur before every test to ensure proper sensor performance. Different patterns of sequencing also provided additional insight: short, repetitive chronoamperometry bursts provided different (yet still insightful) indications of the target analyte in comparison to a single prolonged chronoamperometry sequence. Furthermore, stacking electrochemical sequences together provided significant advantage over a single sequence most likely due to the fact that pre-step sequences targeting different redox potentials allow the sensor to filter residual analytes to reduce the probability of their interference with the target analyte during the stimulus sequence^29^. Figures S4 and S5 show laboratory tests of the linearity of response for both single and stacked electrochemical sequences respectively. One will notice that the slopes of the linear regression lines through responses obtained from the stacked sequence show much smaller values in comparison to the single sequence. Subsequent tests with a single sequence in Fig. S4 provide successively lower readings at a much faster rate than that of the stacked sequencing, suggesting that a single sequence is not enough to adequately reset the sensor for the next test. In other words, results are much more reproducible when combining multiple electrochemical sequences. The prototype also underwent a significant upgrade in mechanical design. These mechanical changes primarily affected usability and airflow. It was found that the sensor produced a much more reliable response when the vector of airflow approached the sensor at a 30° angle of attack or less. Regulating pressure also turned out to be a critical issue to solve—too little pressure and the airflow would equalize with the ambient air before contacting the sensor while too much pressure caused extremely turbulent airflow or allowed the air to bypass the sensor entirely. Laboratory experiments showed that a better sensor response was produced with airflow pressures of 1 psi or less. Due to these findings, mechanical parts were manufactured to control the airflow according to these constraints and peripheral sensors were later incorporated into the airflow path. Additional software upgrades allowed these peripheral sensors to provide closed-loop feedback on the quality of air contacting the electrochemical sensor when detecting the target analyte.

### Sample collection and clinical study

Sample collection was performed at Cleveland Clinic Abu Dhabi Hospital, UAE from patients (n = 84 unique patients) being investigated for the “Detection of Covid 19 Infections by Means of Volatile Organic compounds (VOC) Profile in Exhaled air”. The study design and experimental protocols were approved by the REC and IRB at Cleveland Clinic Abu Dhabi Institutional Review Board (IRB) number REC A-2020-043, RE 20-050, Approval Date 21 May 2020 under which, the patient consent forms were signed and collected from all subjects. The data obtained was de-identified and used for device performance analysis only. All the methods and study protocols on human subjects were carried out in accordance with the Declaration of Helsinki and followed clinical guidelines and regulations.

### Statistical analyses

All the data analysis and interpretations were done using OriginPro. N = 3 replicates have been used for experimentation throughout the manuscript.

### Ethics declarations

Craig Micklich, Kordel France, Ben Micklich and Dr. Bret Cardwell have a significant interest in SOTECH Health, a company that may have a commercial interest in the results of this research and technology. The potential individual conflict of interest has been reviewed and managed by The University of Texas at Dallas and played no role in the study design; in the collection, analysis, and interpretation of data; in the writing of the report, or in the decision to submit the report for publication. Dr. Shalini Prasad, Dr. Anirban Paul and Ivneet Kaur Banga do not have any competing interests.

## Supplementary Information

Supplementary Information.

Supplementary Video 1.

## Data Availability

Data generated and analyzed during this study is available from the corresponding author upon request.
