# Supplementary material for: E.Co.Tech-electrochemical handheld breathalyzer COVID sensing technology
Source: Sci Rep. 2022 Mar 14;12:4370. doi: 10.1038/s41598-022-08321-x (PMC8919908; doi:10.1038/s41598-022-08321-x)
Supplement: Supplementary file 1 — Supplementary Information. [file 41598_2022_8321_MOESM1_ESM.docx]

**SUPPLEMENTARY INFORMATION**

**E.Co.Tech-Electrochemical Handheld Breathalyzer COVID Sensing Technology**

**Ivneet Banga^1#^, Anirban Paul^1#^, Kordel France^2^, Ben Micklich^2^, Bret Cardwell^2,3^, Craig Micklich^2^, Shalini Prasad^1*^**

**^1^ Department of Biomedical Engineering, University of Texas at Dallas, 800 W Campbell Rd., Richardson, TX, 75080, USA.**

**^2^ Sotech Health 17217 Waterview Pkwy Dallas, TX, 75252, USA**

**^3^ Cleveland Clinic Abu Dhabi, Al Maryah Island, Abu Dhabi**

**^#-^Authors share equal contribution.**

***Corresponding author-shalini.prasad@utdallas.edu**

**
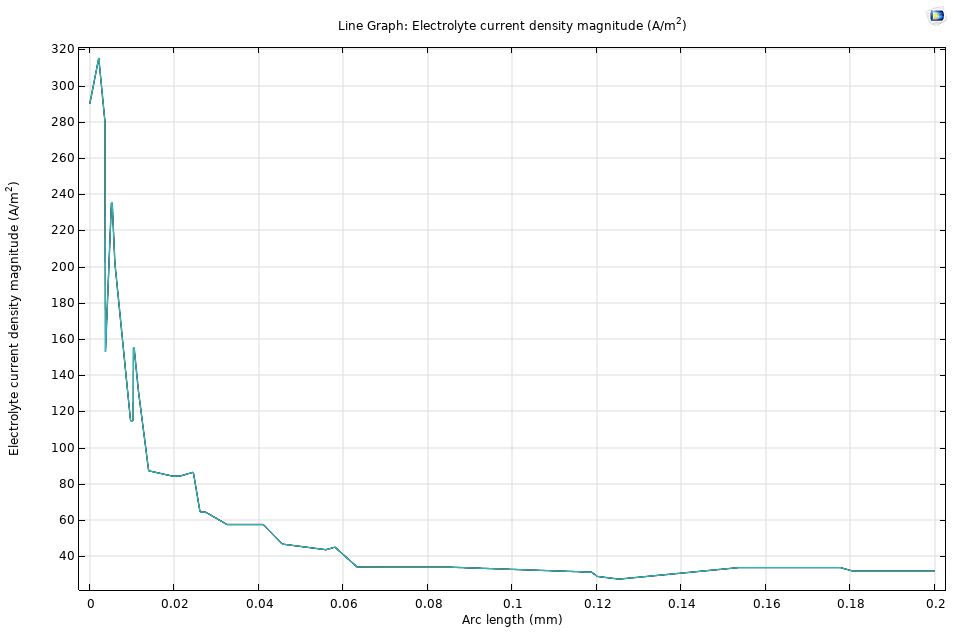
**

**Figure S1-IDE sensor current density graph**


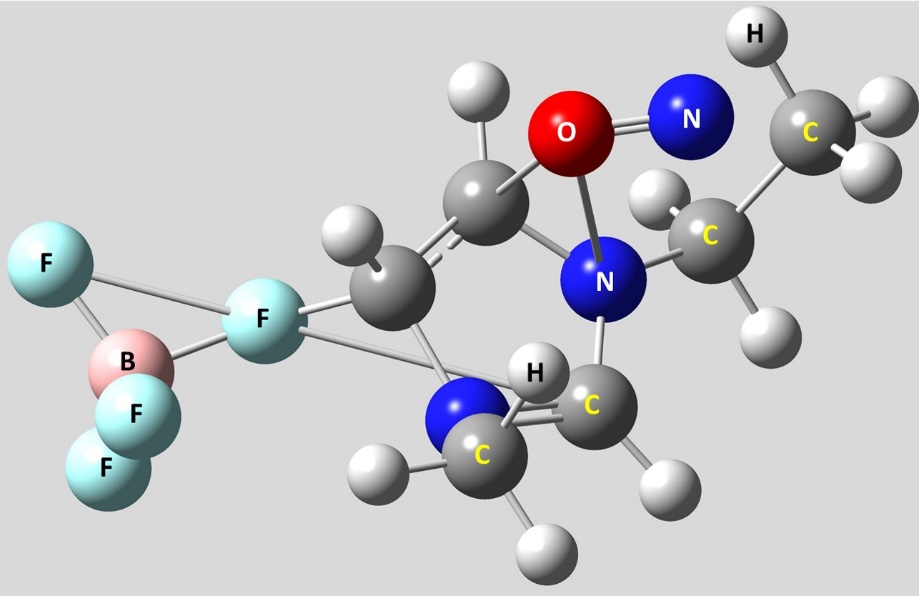


**Figure S2**- optimized structure of EMIM[BF_4­_]

**
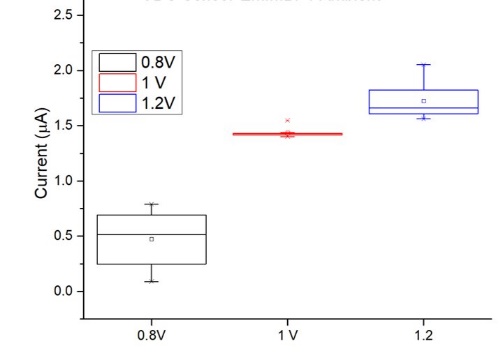
**

**Figure S3-** Electrochemical window optimization of RTIL for gas sensing.

**
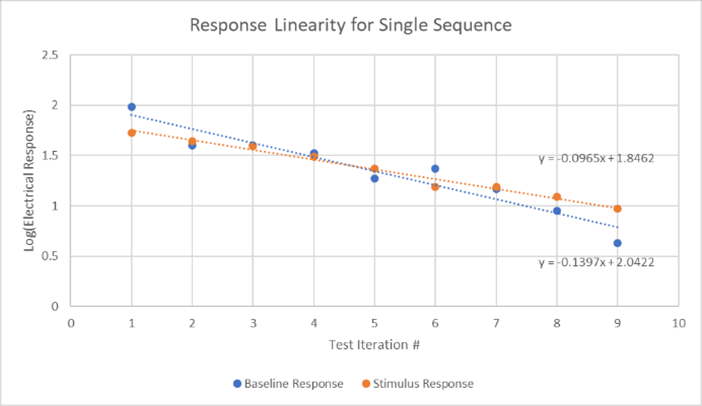
**

**Figure S4-** Sensor response for a single electrochemical sequence


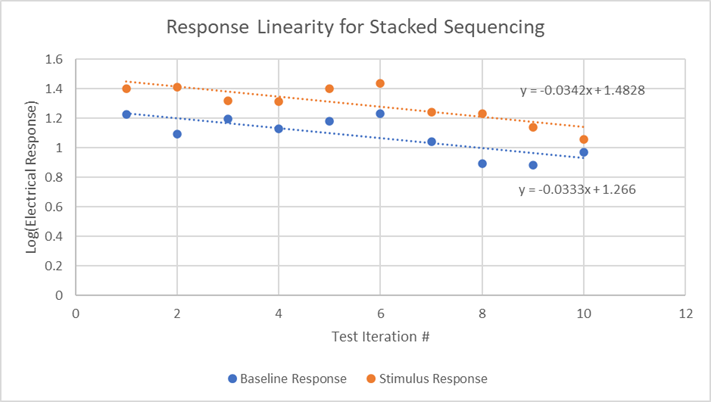


**Figure S5-** Sensor response for stacked electrochemical sequence

| S.No. | Method/Technique | Material | Application | Ref. |
| --- | --- | --- | --- | --- |
| 1 | Impedimetric | SPCE/NPs/nano-Dendroids/GO/Ab probe | COVID detection | ^1^ |
| 2 | Differential Pulse Voltammetry | p-sulfocalix[8]arene (SCX8) functionalized graphene | COVID detection | ^2^ |
| 3  4 | Differential Pulse Voltammetry  Voltammetry | molecularly imprinted polymer with ncovNP  Au micropattern | SARS-CoV-2 nucleocapsid protein (ncovNP)  COVID-19 protein | ^3^  ^4^ |
| 5 | Electrochemical/ Chronoamperometry | [EMIM]BF4 | NO sensing | This work |

Table S1- Table of comparison of different techniques or methods with their application
